# Sugars and Sweet Taste: Addictive or Rewarding?

**DOI:** 10.3390/ijerph18189791

**Published:** 2021-09-17

**Authors:** Danielle Greenberg, John V. St. Peter

**Affiliations:** 1 NutriSci Inc., Mt. Kisco, NY 10549, USA; 2 Deptartment of Experimental & Clinical Pharmacology, University of Minnesota, Minneapolis, MN 55455, USA; stpet003@umn.edu

**Keywords:** food addiction, sugar addiction, food cues, reward, obesity, food behavior, binge eating, eating addiction

## Abstract

The notion of food “addiction” often focuses on the overconsumption of sweet tasting foods or so-called sugar “addiction”. In the extreme, some have suggested that sugar and sweet tastes elicit neural and behavioral responses analogous to those observed with drugs of abuse. These concepts are complicated by the decades long uncertainty surrounding the validity and reproducibility of functional magnetic resonance imaging (fMRI) methodologies used to characterize neurobiological pathways related to sugar and sweet taste stimuli. There are also questions of whether sweet taste or post-ingestion metabolic consequences of sugar intake would lead to addiction or excessive caloric intake. Here, we present a focused narrative review of literature related to the reward value of sweet taste which suggests that reward value can be confounded with the construct of “addictive potential”. Our review seeks to clarify some key distinctions between these constructs and questions the applicability of the addiction construct to human over-eating behaviors. To adequately frame this broad discussion requires the flexibility offered by the narrative review paradigm. We present selected literature on: techniques used to link sugar and sweet tastes to addiction neurobiology and behaviors; sugar and sweet taste “addiction”; the relationship of low calorie sweetener (LCS) intake to addictive behaviors and total calorie intake. Finally, we examined the reward value of sweet tastes and contrasted that with the literature describing addiction. The lack of reproducibility of fMRI data remains problematic for attributing a common neurobiological pathway activation of drugs and foods as conclusive evidence for sugar or sweet taste “addiction”. Moreover, the complicated hedonics of sweet taste and reward value are suggested by validated population-level data which demonstrate that the consumption of sweet taste in the absence of calories does not increase total caloric intake. We believe the neurobiologies of reward value and addiction to be distinct and disagree with application of the addiction model to sweet food overconsumption. Most hypotheses of sugar “addiction” attribute the hedonics of sweet foods as the equivalent of “addiction”. Further, when addictive behaviors and biology are critically examined in totality, they contrast dramatically from those associated with the desire for sweet taste. Finally, the evidence is strong that responses to the palatability of sweets rather than their metabolic consequences are the salient features for reward value. Thus, given the complexity of the controls of food intake in humans, we question the usefulness of the “addiction” model in dissecting the causes and effects of sweet food over-consumption.

## 1. Introduction

The dramatic global increase in the prevalence of overweight and obesity and associated severe negative health consequences has led to an increased focus on underlying mechanisms and possible control measures. A recent trend has been the concept of food “addiction” with a particular emphasis on “addiction” to sugar or to sweet taste. According to neurobiologists, “addictions involve persistent, compulsive, and uncontrolled behaviors that are both maladaptive and destructive” [1,2]. Overeating of palatable foods that inevitably lead to obesity is certainly maladaptive and is often characterized as both persistent and compulsive. These similarities do not, however, mean that the processes underlying overeating and drug addiction are the same, nor that it is appropriate to characterize overeating as food “addiction”. The headline that has never been seen is: “Restaurants and food stores now lock away sugar packets to stop stealing by sugar addicts.” Drug addicts often steal money or valuables from relatives, neighbors, and close friends to support their habits. Drug addicts also engage in other self-destructive behaviors such as sharing dirty needles [2]. Increased illegal behaviors are not seen in people with overweight or obesity. Rather, elevated BMI is associated with a significantly lower risk for committing crimes leading to arrest [3]. 

Multiple neuroendocrine systems are involved in the control of food intake and obesity, including multiple interactions between signals arising from the mouth, the gut, the brain, and adipose tissues [4]. For example, there are specific controls of body weight arising from peptides from fat cells such as adiponectin that are not subsumed by any models of addiction [5]. It has long been known that satiety signals from vagally mediated gastric distention is critical to the control of food intake [6,7]. Oesch et al. (2006) quantified the impact of gastric distention on food intake noting that “The factors that regulate food intake and satiation are complex. Food intake is regulated by chemical and mechanical factors acting in concert to produce sensations of satiety” [8]. In addition, various gut peptides such as CCK interact with gastric distension to effect changes in food intake [4,6,9,10,11]. To date, these controls have not been implicated in models of addiction [12,13,14,15]. 

There are certain assumptions that explain use of the concept of food “addiction”, namely that certain foods, such as those that taste sweet or combine sweet taste with fats, stimulate parts of the brain that are also stimulated by addictive drugs, and that individuals can develop behaviors, such as binge eating that may mimic behavior patterns seen in substance-use disorders [16]. It should be noted that food addiction is not currently a validated concept as it has not been included in the Diagnostic and Statistical Manual of Mental Disorders (DSM)—V, the leading classification system for diagnosing mental diseases [17]. Additionally, the American Psychiatric Association notes that, despite the similarities between eating disorders and drug abuse, and evidence of the involvement of brain reward circuits in both conditions, “the neurobiology of binge eating and drug addiction are not the same” [17]. 

Despite the scientifically accepted differences between substance abuse disorders and eating disorders, the concept of food “addiction” has been widely adopted by some scientists and the lay press [13,18,19,20,21,22,23,24,25,26,27,28,29,30,31,32]. One basis for this concept comes from a limited number of animal studies [6]. The other group of studies used to support the concept of food “addiction” are based on functional magnetic resonance imaging (fMRI) studies. Here, we review some of the history and development for the concept of food “addiction”. Others have noted the lack of scientific evidence substantiating an addiction model that foods can act to disinhibit food consumption control centers [15,33]. Further, the exact nature of the addictive stimulus found in food(s) has not been characterized [15,33].

We believe a more useful and robust scientific framework to characterize and investigate overconsumption of sweet tasting and other palatable foods is that of reward value and satiating potency rather than the more limited aspects of “addiction”. Given the more inclusive neurobiology of reward value and satiating potency, scientific explorations of responses to these human biology paradigms may offer more avenues for intervention to prevent overweight and obesity than does an “addiction” model. Human hedonic processes have been recognized as unique for over 50 years [34]. The necessity of distinguishing between habit and palatability as separate determinants of food choice was first pointed out by Paul Thomas Young [35]. Confusing these fundamental food choice concepts and related underlying neurobiology with “addiction” confounds scientific study design and potential interventions addressing overeating and obesity. Here, a selected narrative review that focuses on differentiating between the concepts of hedonic reward and addiction is presented. We believe, as noted by Collins and Fauser [36], the narrative review can be most appropriate when applied to discussion of clinical concepts. Supporting this narrative, we applied the search terms “sweet taste reward and food addiction” and “hedonics of sweet taste and food addiction” to PubMed and Google Scholar.

## 2. Insights from Animal Studies

The reward characteristics of sweet taste from either nutritive or non-nutritive sources has long been known [37]. In order to determine whether foods act like drugs of abuse in leading to addiction, it is necessary to explore how drugs such as opiates act to evoke addiction. As reviewed by Roy Wise as early as 1987, behavior controlled by drugs appears to mimic behavior controlled by natural rewarding substances [38]. Drugs of abuse can recruit similar controls as are seen by any rewarding substance due, at least in some cases, to central drug actions in specific brain circuitry primarily mediated by dopamine [39,40]. The facilitatory interactions between drugs of abuse and rewarding brain stimulation from palatable foods suggest common mechanisms, but this does not prove that the behaviors are identical. The mechanisms of action for drug addiction have been identified for at least some drugs of abuse and they mostly center on dopamine pathways and involve the amygdala and other structures mediating dopamine reward [1,14,41,42,43]. Exogenous drug reward has often been shown to depend on endogenous neurotransmitters [44]. Both agonists and antagonists for these neurotransmitters either enhance or counter respectively the effects of exogenous opiates [45] and other drugs of abuse [46]. 

Claims about sugar “addiction”, however, are based largely on findings from relatively few animal studies. Early experiments with selective d-1 and d-2 dopamine antagonists in sham-feeding animals showed that reward from both sweet and dietary fat stimuli were mediated by dopamine receptors [47,48,49,50]. Sham feeding was used to isolate the orosensory stimulation by sucrose or corn oil while minimizing post-ingestive effects. Dopamine antagonists decreased intake in a way that showed they were decreasing the reward value of sweets or fats [48,49,51]. Other experiments using microdialysis techniques showed that dopamine released from the nucleus accumbens was necessary for the normal rewarding effect of sucrose [1,45,49,52,53]. Early work also demonstrated that similar increases in dopamine were found subsequent to the administration of cocaine [53]. 

Other regions of the brain in the mesolimbic and mesocortical dopamine systems have also been shown to be important for motivational function including those in the amygdala and hippocampus [40]. Many studies in animals with a focus on the dopamine system underlie the hypothesis that the abuse potential from sweet or fatty foods is analogous to abuse seen in drug addiction. While both foods and drugs have shared brain reward pathways, food intake is extensively controlled by numerous other systems (i.e., feedback from adipose tissue, gastric distension, etc.) not currently recognized as related to addiction. Coincident shared brain reward pathways have been extended by some to state the sugar “addiction” hypothesis which assumes that sweetness from sugars or non-nutritive sweeteners leads to overeating and eventually obesity [18,25,54,55,56,57,58,59,60,61,62]. In these experiments, rats are given intermittent access to sweet tastes followed by absence of these stimuli which lead to elevated consumption of the sweet solutions. Brain dopamine, however, was stimulated in patterns similar to those elicited by drugs of abuse [42]. Weight gain was not typically observed [24,25,26,27,28,29,30,31,32,33]. As noted by Markus et al. [63] “…these apparent similarities between sugar and drugs on dopamine have given rise to the sugar addiction hypothesis of binge eating. However, acting on similar mechanisms does not necessarily indicate equal parameters of addiction” [63]. 

More recent work in mice has explored the role of systems beyond dopamine reward in the control of food intake. This work clearly suggests both separation and commonality between addiction and controls of food intake. For example, dopamine receptor blockade had no effect on food reduction elicited by melanocortins, nor did dopamine receptor blockade reduce the stimulation of food intake induced by ghrelin [4,64,65,66]. Alternatively, blocking dopamine receptors did attenuate the reduction of food intake induced by leptin, suggesting leptin may evoke its effects through reward mechanisms [65]. Glucagon-like peptide-1 (GLP-1) agonists have recently been used in the treatment of both type 2 diabetes and obesity [67,68,69]. It should be noted that GLP-1 also is implicated in mediating the effects of alcohol intake. The GLP-1 analogue exendin-4 attenuates the ability of alcohol to activate the mesolimbic dopamine system, as measured by alcohol induced locomotor stimulation, and also attenuates dopamine release in the nucleus accumbens in mice [70].

Further demonstrating the separation and commonality between addiction and food reward constructs, it is important to note that multiple systems are involved in the control of food intake and obesity. These include multiple interactions between signals arising from the mouth, the gut, the brain, as well as white, beige, and brown adipose tissues [4]. There are signals that tend to increase food intake, such as ghrelin, neuropeptide Y (NPY), and agouti-related protein (AgRP), and those that decrease food intake, such as cholecystokinin (CCK), pancreatic glucagon, amylin, peptide YY (pYY), and leptin [4,71,72,73,74,75]. There are direct neural signals, such as those arising from gastric distention that inhibit food intake through vagal afferents, and these signals interact with taste signals in the hindbrain specifically at the nucleus tractus solitarius and the area postrema [74,76,77,78,79,80,81]. In addition, recent studies show that adipose tissue itself is not only an energy storage depot, but also an endocrine organ that secretes hormones such as adiponectin that is critical in maintaining energy balance [5,82,83]. There do not exist, to date, any models of addiction that involve all the systems known to be active in the control of food intake and the development of obesity. As noted by Fletcher [15], “The application of the term “food addiction” in humans is based on a set of features, held to resemble substance addictions. It carries the claim that this resemblance occurs because certain foods have effects on the brain comparable to those of addictive drugs.” He suggests that both of these claims are in error because the first is that the main features of substance addiction do not translate to food and consumption. He also rejects the notion that foods have the same pharmacological effects on the brain which he notes lacks strong and convincing evidence [15].

Reward models are physiologic constructs that more completely incorporate and describe the known neurobiology of food intake controls. This level of complexity is necessary in order to promote the most robust avenues for investigating and developing interventions that may impact overweight and obesity. Berridge [84] has stated this hypothesis very well. He suggests that reward subsumes both “liking” (pleasure/palatability) and “wanting” (appetite/incentive motivation) which are separate and distinct, that they have separable neural substrates, and that these substrates are mediated by distinct and separate parts of the brain. He further notes that various experimental manipulations can enhance reward or produce aversion, but none of these require an “addiction” model. 

## 3. Food “Addiction” in Humans

Over the past 20 years there have been numerous studies of the concept of food/sweet taste “addiction” in humans. Review articles both support and counter the concept [12,15,21,24,26,30,31,85,86,87,88,89]. Human dietary studies do not, however, support the conclusion that a particular micronutrient or taste such as sugar or low calorie sweeteners cause binge eating and/or weight-gain more so than other food sources [90,91,92,93,94]. In fact, those who consume low calorie sweeteners tend to eat fewer calories and fewer carbohydrates, suggesting that consuming sweet tasting substances do not lead to overeating or other addictive behaviors [92,95]. One strategy for identifying if the so-called addictive properties of foods are present involves determination of whether certain DSM-V [17] criteria for substance use disorder are met. Brownell and his colleagues developed the Yale Food Addiction Scale (YFAS) that uses some DSM criteria for substance disorders to quantify symptoms of ‘addiction-like eating’ for highly palatable, energy-dense foods [22,96]. As mentioned previously, it is important to note that the American Psychiatric Association examined these findings and declined to include “food addiction” as a diagnosable disorder in the DSM-V [17].

Many investigators have used fMRI to link addiction behavioral responses with biochemical mechanisms observed between body weight and highly palatable sugars, sweet taste or fats [22,23,97,98,99,100] The fMRI test measures brain activity by detecting changes associated with blood flow. Some studies used images of foods rather than actual ingestion thus responses may or may not reflect what would occur when stimuli are consumed [101]. Other fMRI studies used odors of foods rather than consumption [98]. More recently, food tasting has been used but few fMRI studies examine responses to solid foods and often these foods are not those that subjects report as most preferred [99]. The brain circuits activated in these human fMRI studies show brain circuitry similar to those involved in both food reward and drugs of abuse in rodents [37,38,39,42,45,49]. Merely activating the same reward circuitry does not necessarily mean that the full complement of behaviors elicited by drugs of abuse also exist for palatable foods. 

There have been few clinical studies testing whether foods high in sugars lead to obesity and cause ‘addiction-like’ problems that meet DSM criteria for substance dependence. One study by Markus et al. [63] found that while there was almost always some characteristic (95% of subjects) that was related to DSM criteria and related to self-reported eating behavior. This, however, was not necessarily related to consumption of sugary foods. Sugary foods contributed minimally to ‘food dependence’ (>5%) nor were these sugary foods associated with increased risk for weight gain. Rather, they found that the reward value and energy density of foods as well as environmental determinants were most important in promoting excessive energy intake [17]. Whether there is enough evidence for explaining the variance in obesity etiology by food “addiction” or sugar “addiction” remains unclear [30,33]. Further research needs to focus on more than neural circuitry and responses to clinical instruments to include the wide variety of behaviors that are seen in addiction to drugs of abuse.

## 4. Issues with fMRI Studies

There is general acceptance of fMRI studies as a means of assessing brain physiological responses to external stimuli. There are qualifications for interpreting these results that are often forgotten. What fMRI actually measures is increased blood flow to a given area of the brain and while this is widely accepted as indicating increased activity in that anatomical region, fMRI cannot indicate what neurotransmitters are being released. Since some neurotransmitters are excitatory and some inhibitory, increased blood flow cannot be assumed to be excitatory in nature. It has been noted that while fMRI can be a powerful and versatile tool for the study of neural substrates of cognitive and perceptual functions, exactly how it is coupled to the underlying neurophysiology, and how this coupling reflects underlying processes that can be both inhibitory an excitatory and can involve multiple neurotransmitters and receptor characteristics. The interpretation of fMRI is also complicated by factors within the same cortical region, multiple evoked and induced oscillatory effects, and any or all of these may be reflected in the overall fMRI signals [102]. There is little evidence that fMRI can detect differences between excitatory and inhibitory synapses [103]. Others have noted that “The fMRI signal is too easily affected by many different endogenous and exogenous factors that are difficult to control. Even with standardized acquisition and analysis protocols, substantial and clinically irrelevant variations in individual fMRI results will be still present” [104]. 

In addition, recently, Eklund et al. [105] found that many of the statistical measures used in fMRI experiments had not been validated. Eklund’s findings questioned the validity of a large number of fMRI studies and suggested that there may be widespread misinterpretation of weakly significant neuroimaging results. While this lack of statistical validation does not nullify the results of the many published fMRI studies on responses to foods or drugs of abuse, these findings do suggest that extreme caution should be used in assessing the results of these studies. 

## 5. Conclusions

### Palatability and Reward A Better Framework

Food has both homeostatic and hedonic components, which make it a potent natural reward. It is necessary to consume food for life. Drugs of abuse may interact with some of the same reward pathways that were evolutionarily advantageous to promote food intake. Attributing the negative addictive qualities of drugs of abuse to foods may be appealing but does not necessarily lead to a greater understanding of interventions that are likely to be effective in reducing excessive food intake. Within an addiction construct, specifically focusing on particular nutrients such as sugars or any stimulus of sweet taste and then suggesting avoiding these foods is not likely to be a useful strategy for dealing with the present rates of overweight and obesity.

The drive to eat, and specifically the drive to eat palatable foods, is one component of appetite control, but it is not the only one. Homeostatic mechanisms involved in responding to energy expenditure along with environmental cues are equally if not more important [106]. The reward value of foods need to be considered within the framework of energy balance. Scientific investigations incorporating the more complete framework of food reward value are likely to be of greater use in addressing the very real problems of obesity and the overconsumption of foods with low nutrient density. The use of colloquial terminology, such as food or sugar “addiction”, within scientific publications distracts from the purpose of clear, concise, and objective sharing of scientific observations related to the control of food intake.
